# Ultrasonographic assessment of splenic volume at presentation and after anti-malarial therapy in children with malarial anaemia

**DOI:** 10.1186/s12936-015-0741-0

**Published:** 2015-05-28

**Authors:** Moses Laman, Susan Aipit, Cathy Bona, Peter M. Siba, Leanne J. Robinson, Laurens Manning, Timothy M. E. Davis

**Affiliations:** School of Medicine and Pharmacology, University of Western Australia, Fremantle Hospital, PO Box 480, Fremantle, 6959 WA Australia; Papua New Guinea Institute of Medical Research, Madang, Papua New Guinea; Department of Microbiology, Division of Veterinary and Biomedical Sciences, James Cook University, Townsville, Australia; Infection and Immunity Division, Walter and Eliza Hall Institute, Parkville, VIC Australia; Department of Medical Biology, University of Melbourne, Melbourne, VIC Australia

**Keywords:** Malaria, *Plasmodium falciparum*, Anaemia, Splenic volume, Children

## Abstract

**Background:**

Splenic enlargement is a component of the host response to malaria and may also influence the genesis and progression of malarial anaemia. Few cross-sectional and no longitudinal studies have assessed the relationship between splenic volume measured ultrasonographically and haemoglobin concentrations in children with malaria.

**Methods:**

Fifteen Papua New Guinean children with severe malarial anaemia (SMA; haemoglobin <50 g/L) and ten with moderate malarial anaemia (MMA; 51–99 g/L) were recruited. The SMA patients were given intramuscular artemether followed by oral artemisinin combination therapy (ACT), and were transfused one unit of packed cells 0.3-4.0 days post-admission. The MMA patients were treated with ACT. Splenic enlargement (Hackett’s grade, subcostal distance and ultrasonographically determined volume) and haemoglobin concentrations were measured on days 0, 1, 2, 3, 7, 14, 28, and 42.

**Results:**

Associations between Hackett’s grade, subcostal distance and splenic volume were modest (r_s_ ≤ 0.62, *P* <0.001). Baseline splenic volume was not associated with age or haemoglobin (*P* ≥0.90). Mean splenic volume had fallen by approximately 50 % at day 14 in children with MMA (*P* ≤0.011 *vs* days 0, 1 and 2), but there was no change in the SMA group (*P* ≥0.30). There was no change in haemoglobin in the MMA group during follow-up but a rise in the SMA group to day 7 (*P* ≤0.05 *vs* days 0, 1, 2, and 3) which paralleled the packed cell volume transfused.

**Conclusions:**

Clinical assessment of splenomegaly is imprecise compared with ultrasonography. Serial splenic volumes and haemoglobin concentrations suggest that the spleen does not influence post-treatment haemoglobin, including after transfusion.

## Background

**Fig. 1:**
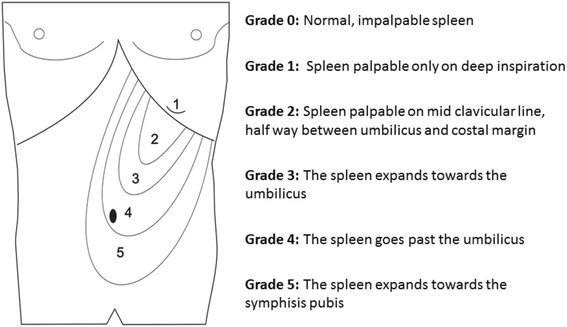
Hackett’s grading system for palpable splenomegaly

Splenomegaly is one of the oldest known clinical manifestations of malaria, having been recognized before the discovery of the *Plasmodium* parasite [1]. Spleen size is conventionally graded according to Hackett’s system [2] with a score ranging from zero (no palpable spleen) to five (massive enlargement extending towards the umbilicus; see Fig. 1), but the vertical distance in centimetres (cm) from the lower costal margin in the mid-clavicular line has also been used in studies of malaria [3]. The rate of palpable splenomegaly can provide an indication of the intensity of malaria transmission in endemic areas when blood smears are unavailable [2, 4].

In patients with malaria, clearance of both parasitized and non-parasitized red blood cells results in splenic enlargement [5, 6] which in turn contributes, along with intravascular haemolysis and dyserythropoiesis, to anaemia [7–10]. Animal studies also show the pathophysiological importance of splenic enlargement, with explanted and post-mortem spleen sizes associated with the severity of anaemia complicating malaria [11, 12]. The spleen is also an important site for pathogen-specific adaptive and maladaptive T and B cell immune responses. At its most extreme, aberrant T-cell immune regulation of effective humoral responses can lead to the massive splenic enlargement seen in hyper-reactive splenomegaly syndrome, a major public health problem in some populations which is associated with significantly increased mortality.

The complex three-dimensional shape of the spleen and its position in the left upper quadrant of the abdomen complicate accurate assessment of splenic volume. Since Hackett’s grading or simple uniaxial measurement may have insufficient sensitivity, ultrasonography has been used as a more accurate alternative to clinical assessment in parasitic diseases such as schistosomiasis [13]. In the case of malaria, one small paediatric study showed that spleen size by palpation significantly overestimated the true incidence of splenomegaly as measured by ultrasound [14]. Another study involving largely adults questioned the value of splenic ultrasound as an aid to diagnosis and did not find that ultrasonographically detected splenic enlargement was associated with indices of severity [15]. By contrast, children with severe malaria anaemia (SMA; haemoglobin concentration <50 g/L) in a second cross-sectional paediatric study had higher splenic volumes adjusted for total body surface area than those with cerebral malaria, and a low splenic volume was associated with subsequent mortality [16]. There have, however, been no studies that have compared serial changes in splenic volume after treatment assessed by ultrasound to both simpler clinical measurements of spleen size and related outcomes such as the development of malarial anaemia. The spleen generally regresses in size within days to weeks after anti-malarial treatment [17], but blood transfusion as part of the management of SMA may promote persistent splenic enlargement which could, in turn, attenuate the benefits of transfusion [18].

In malaria-endemic areas of Papua New Guinea (PNG), malarial anaemia associated with splenomegaly is a common clinical presentation. Despite a declining incidence of malaria, 13 % of healthy asymptomatic PNG children have splenomegaly [19], while anaemia, including SMA, is common and multifactorial [20]. The aim of the present study was to i) compare the traditional methods of splenic size assessment with ultrasonographic volumetric measurement; and, ii) evaluate serial changes in spleen size and their haematological consequences in children with SMA and moderate malarial anaemia (MMA).

## Methods

### Study sites, patients and approvals

Children with SMA were enrolled between March 2009 and May 2010 as participants in a prospective observational study of severe paediatric illnesses that was conducted at Modilon Hospital on the north coast of mainland PNG. The clinical and laboratory characteristics of these patients have been described previously [20]. In brief, they were aged one to ten years and had *Plasmodium falciparum* and/or *Plasmodium vivax* on light microscopy of a peripheral blood smear together with severe anaemia. Standardized case report forms containing demographic and clinical information were completed by trained research nurses. Clinical management, including blood transfusions, were in accordance with World Health Organization (WHO) [21] and PNG national guidelines [22]. Intramuscular artemether was given at an initial dose of 3.2 mg/kg of body weight, followed by daily doses of at least 1.6 mg/kg until oral artemisinin combination therapy (ACT) could be tolerated. Daily clinical assessments were performed in hospital. Home visits were conducted at pre-specified time-points after discharge.

Children with MMA (haemoglobin 51–99 g/L) were recruited at Mugil Health Centre located approximately 50 km from the township of Madang between April 2011 and June 2012. Screening for MMA was either part of assessment for eligibility in a randomized trial of ACT if the children were aged < five years [23] (none of the present children met the inclusion criteria for the clinical trial) or it was performed during routine assessment of older children presenting with fever. Inclusion criteria for the present study were i) age between one and ten years; ii) *P. falciparum* and/or *P. vivax* on peripheral blood smear; and, iii) no clinical or laboratory evidence of severe malaria or another infection. All children with MMA were treated with ACT (artemether-lumefantrine) in accordance with the PNG standard treatment guidelines [22].

The study was approved by the PNG Institute of Medical Research Institutional Review Board and the Medical Research Advisory Committee of the PNG National Health Department (MRAC No. 11.07). Written informed consent was obtained from a parent or guardian of each study participant.

### Clinical and laboratory procedures

All children were assessed on days 0, 1, 2, 3, 7, 14, 28, and 42. At baseline, 500 μL venous blood was collected into Microtainer® tubes (Becton Dickenson, Franklin Lakes, USA) containing 0.39 mg disodium edetate and a full blood count was performed using a Coulter counter (ACT3 diff PAK, Beckman Coulter, Brea, USA). At each subsequent follow-up visit, 250 μL blood was collected by finger prick for Coulter counter haemoglobin measurement and malaria microscopy. In children with SMA, an additional 1–3 mL of blood was collected for bacterial culture in Bactec^TM^ Peds Plus^TM^/F bottles (Becton Dickinson) and incubated using an automated Bactec^TM^ system. For malaria microscopy, standard procedures were used [24, 25]. Giemsa-stained thick blood smears were examined and parasitaemia quantified independently by two skilled microscopists, with discrepancies adjudicated by a senior microscopist.

**Fig. 2:**
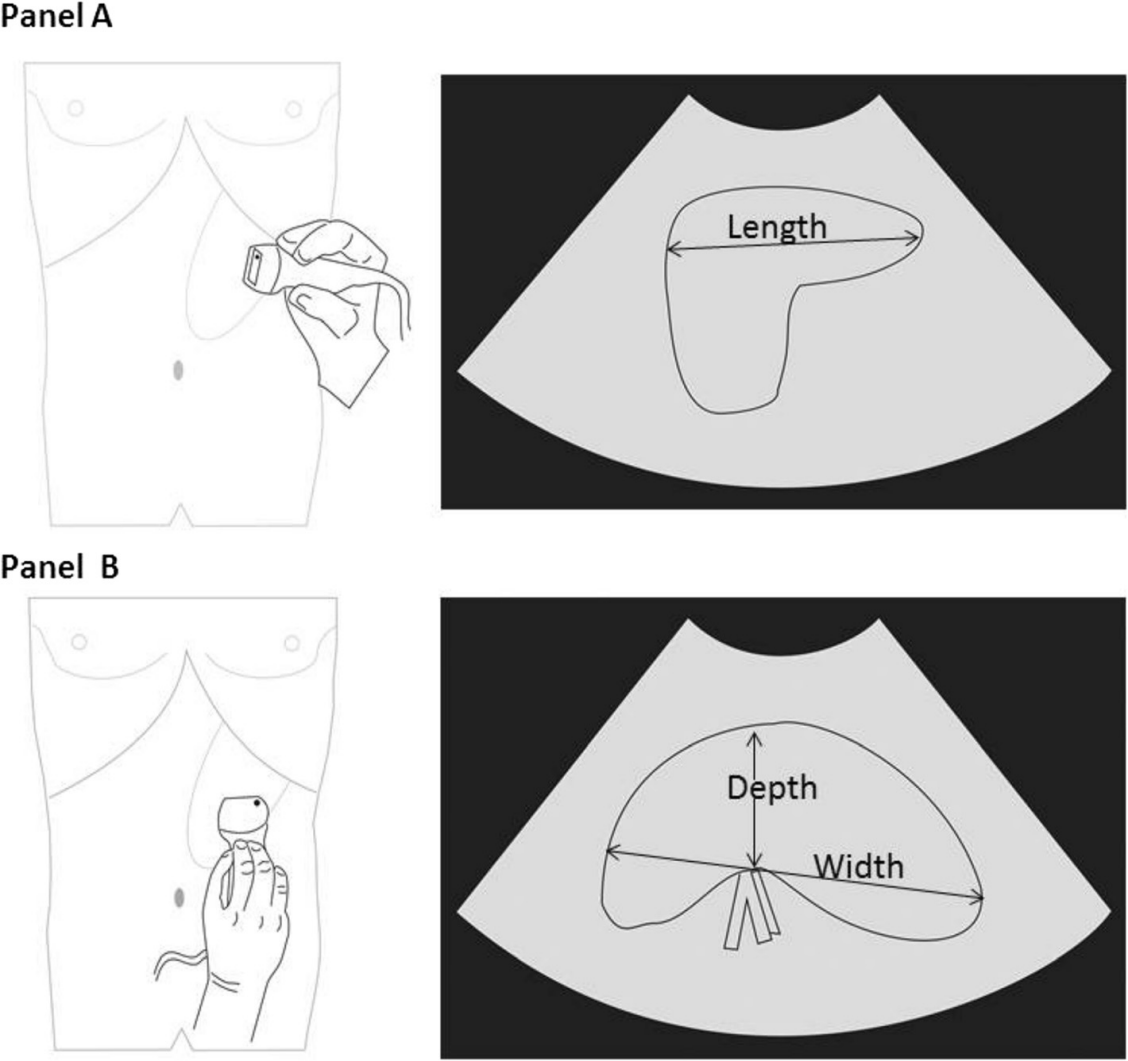
Assessment of splenic volume using ultrasonography. Panel **a** illustrates measurement of spleen length in the longitudinal view. Panel **b** shows measurement of width and thickness on the transverse view

Splenic size was assessed by palpation at each visit with children in the supine position. Hackett’s grading was determined in each case (see Fig. 1) and a tape measure was used to determine the distance in cm from the lower costal margin to the tip of the spleen in the mid-axillary line. Ultrasonography in each SMA and MMA patient and at each time point was performed using a MicroMaxx Ultrasound System (SonoSite, Brookvale, New South Wales, Australia) by the study clinician (ML) or one of two trained nursing officers (SA and CB) with the patient in the right recumbent position. These study staff were all blind to the results of prior splenic volume assessments and were not allocated systematically to individual patients or time points. The spleen length (L), width (W) and depth (D) were assessed using with the probe in the longitudinal axis (L) and transverse axis (W and D; see Fig. 2). With parental assistance, valid images in each dimension were obtained from the majority of children at each time-point. Splenic volume in cm^3^ was calculated from the formula 0.524 × L (cm) × W (cm) × D (cm) where L, W and D were obtained from the best single image recorded [26, 27]. The within- and between-operator variability in splenic volume has proved to be acceptably low in other clinical contexts, including studies involving children and those using a portable ultrasound machine [28–31].

### Data analysis

The computer package IBM SPSS Statistics 20 (IBM Corporation, Somers, NY, USA) was used for statistical analysis. Data are summarized as mean ± SD or median (inter-quartile range). Two-sample comparisons were by Mann–Whitney *U*-test for continuous data and by Fisher’s exact test for proportions. Bivariate associations between variables were assessed using Spearman rank correlation co-efficient (r_s_). For serial splenic volume and haemoglobin data, missing data were imputed up to a maximum of two values per patient using linear interpolation or last observation carried forward. Differences between groups at each time-point were assessed using generalized linear mixed-effect models with gamma regression (given the skewed nature of the splenic volume data) and Bonferroni correction for pair-wise comparisons. A two-tailed significance level of *P* <0.05 was used throughout.

## Results

### Baseline patient characteristics

**Fig. 3:**
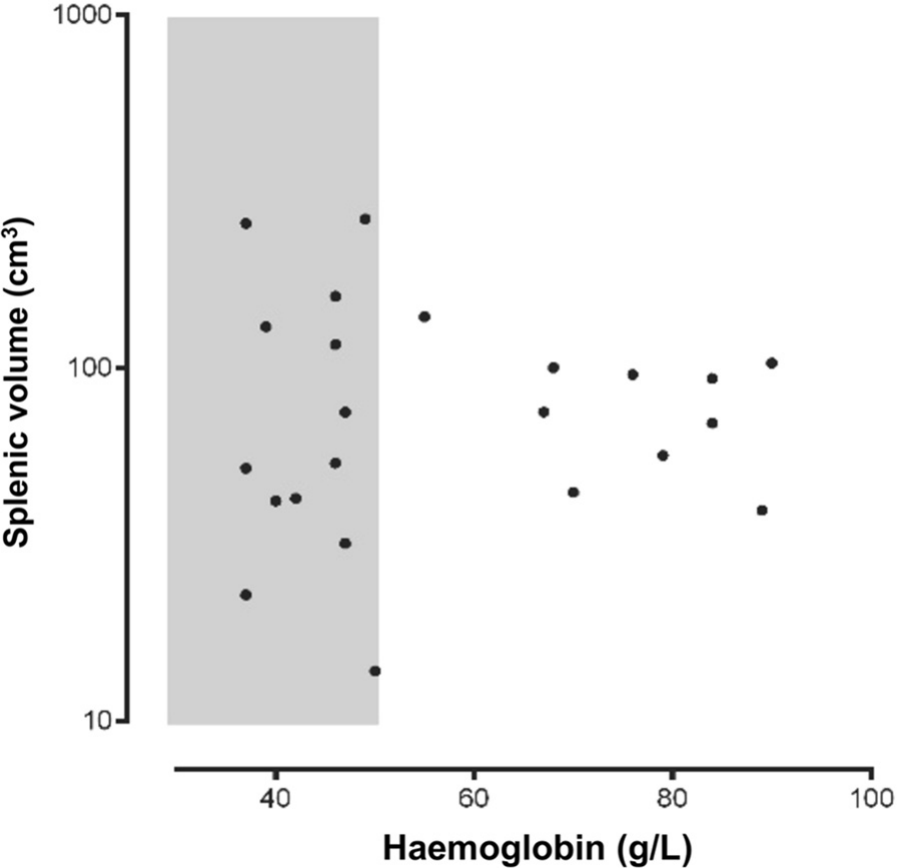
Baseline splenic volume measured by ultrasound plotted against haemoglobin concentration at study entry. The shaded area represents severe malarial anaemia (haemoglobin <50 g/L). Two children with SMA did not have valid baseline splenic volume measurements

Ten children with MMA and 15 with SMA were recruited. Their baseline characteristics are summarized in Table 1. All had *P. falciparum* mono-infections by expert microscopy at the time of enrolment, consistent with the greater transmission, and stronger association with anaemia, of *P. falciparum* vs *P. vivax* in the study area [20, 24]. Children with SMA were significantly younger than those with MMA, and they had a higher pulse rate, total white blood cell count and platelet count. At baseline, splenic volumes measured by ultrasound ranged from 14 cm^3^ to 265 cm^3^. There was no significant difference between the two patient groups in splenic volume, length below the left costal margin or Hackett’s grade (*P* ≥0.50; see Table 1). Baseline splenic volume was not associated with haemoglobin concentration (r_s_ = 0.02; see Fig. 3) or age (r_s_ = −0.03; *P* ≥0.90 in each case).

**Table 1:** Baseline clinical and haematology data in children with moderate and severe malarial anaemia

	Moderate malarial anaemia	Severe malarial anaemia	*P*-value
	(*n* = 10)	(*n* = 15)	
Age (months)	74 (58–87)	36 (32–49)	0.001
Male sex (%)	50	40	0.47
Axillary temperature (°C)	37.1 (36.3-38.6)	37.6 (36.6-38.0)	0.75
Pulse rate (beats/min)	97 (80–120)	128 (109–135)	0.003
Respiratory rate (breaths/min)	28 (23–33)	28 (28–39)	0.14
Blantyre Coma Score ≤4 (%)	0	20	0.20
Palpable spleen size (cm)	5.5 (3.7-7.0)	4 (2.0-7.9)	0.50
Hackett’s grade	2.5 (2–3)	3.0 (1.0-4.0)	0.93
Splenic volume (cm^3^)	84 (49–102)	54 (36–149)^a^	0.66
Total White cell count (10^9^/L)	6.5 (3.1-9.1)	9.5 (6.5-18.8)	0.032
Lymphocytes (%)	46 (32–56)	40 (21–57)	0.42
Monocytes (%)	11 (7–18)	9.6 (6–12)	0.32
Granulocytes (%)	46 (32–53)	51 (32–72)	0.30
Total platelet count (10^9^/L)	108 (50–221)	126 (43–211)	<0.001
Haemoglobin (g/L)	77 (67–87)	46 (39–49)	<0.001
Red blood cell count (10^12^/L)	2.7 (2.3-3.6)	2.2 (1.3-4.4)	0.42
Haematocrit (%)	22 18–26)	16 (10–25)	0.13
Mean cell volume (fL)	66 (62–77)	63 (55–83)	0.56
Mean cell haemoglobin (pg)	20 (19–24)	22 (19–27)	0.25
Mean cell haemoglobin concentration (g/L)	310 (284–324)	339 (308–380)	0.024
Red cell distribution width	17.5 (14.2-21.0)	19.1 (15.3-30.1)	0.42
*P. falciparum* density (/μL)	5776 (235–33,569)	600 (190–3388)	0.37

^a^Two children in the SMA group did not have a valid splenic volume measurement

Data are medians (interquartile range) or percentages

### Clinical course

There was a rapid clearance of *P. falciparum* parasitaemia in all children with MMA. Nine (90 %) were slide negative within 24 h and all were slide negative by day 3. However, gametocyte carriage persisted up to 14 days in two children (20 %) and up to 28 days in one child (10 %). None of the children with MMA required transfusion, and all were discharged from hospital when they were afebrile and aparasitaemic.

All children with SMA were blood slide negative for malaria within 24 h. Gametocyte carriage persisted up to 28 days in one child. Blood cultures performed in children with SMA were negative for bacterial pathogens in all cases. All children with SMA were transfused one unit of packed cells (300 mL) within four days of admission (median 2.5 (range 0.3-4.0) days). There were no deaths during follow-up in the SMA group.

### Associations between splenic volume and clinical assessment of splenic enlargement

**Fig. 4:**
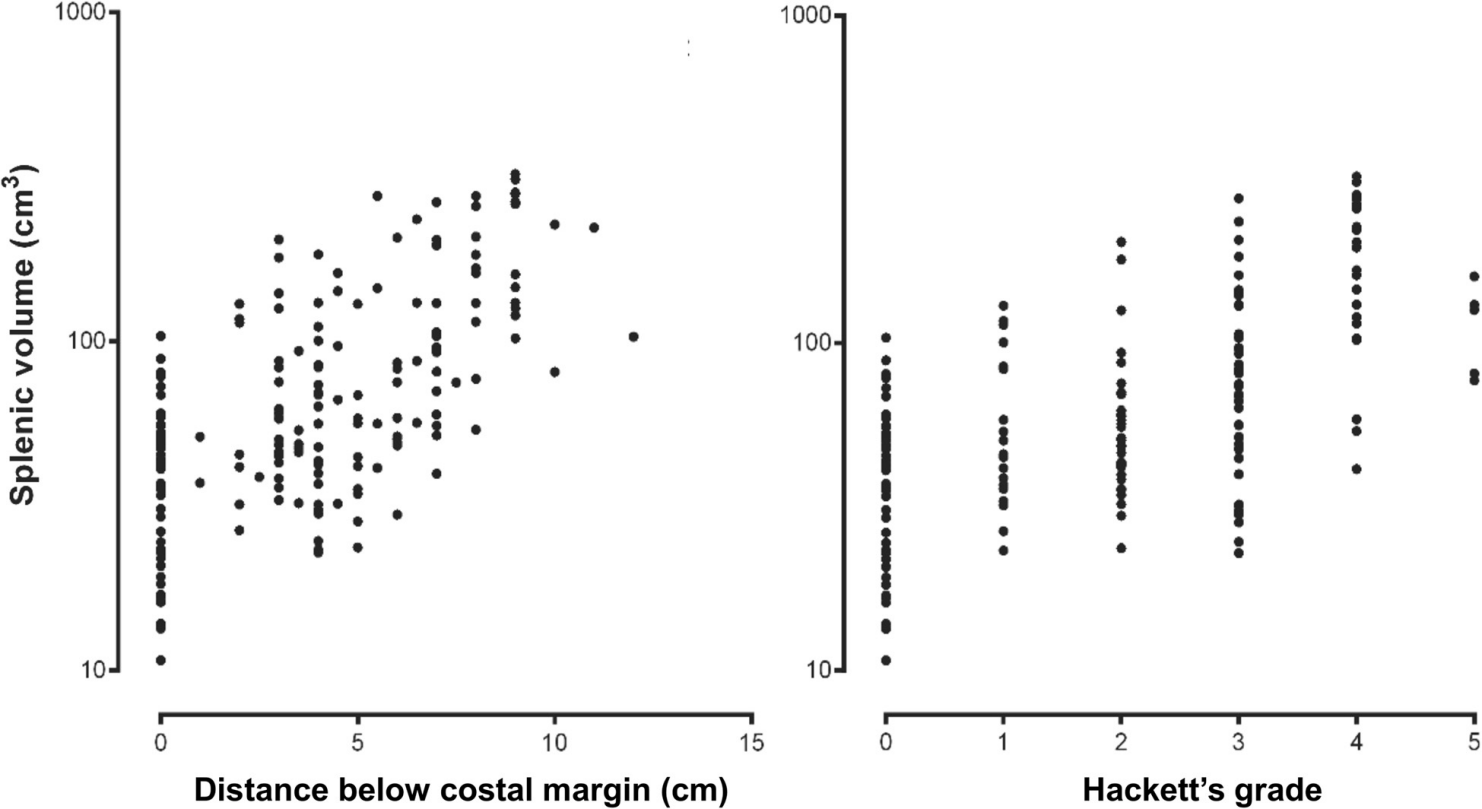
Splenic volume by ultrasound plotted on a logarithmic scale against longitudinal distance in cm below the costal margin (left) and Hackett’s grade (right) in children with moderate or severe malarial anaemia

To assess correlations between the traditional methods of assessing splenic enlargement (distance below the left costal margin and Hackett’s grade), combined measurements from all children were obtained at all time-points. Both the distance below the left costal margin and Hackett’s grade were significantly associated with splenic volume measured by ultrasound (r_s_ = 0.62 and 0.59, respectively, *P* <0.001 in each case; see Fig. 4). However, there was marked variation in both cases with, for example, up to a ten-fold variation in splenic volume at each Hackett’s grade. In those children without palpable splenomegaly, the range of splenic volumes determined by ultrasound (<110 cm^3^; see Fig. 4) was consistent with mean values for normal children aged between one year (42 cm^3^) and ten years (108 cm^3^) as assessed by computed tomography [32].

### Post-treatment changes in splenic volume and haemoglobin

**Fig. 5:**
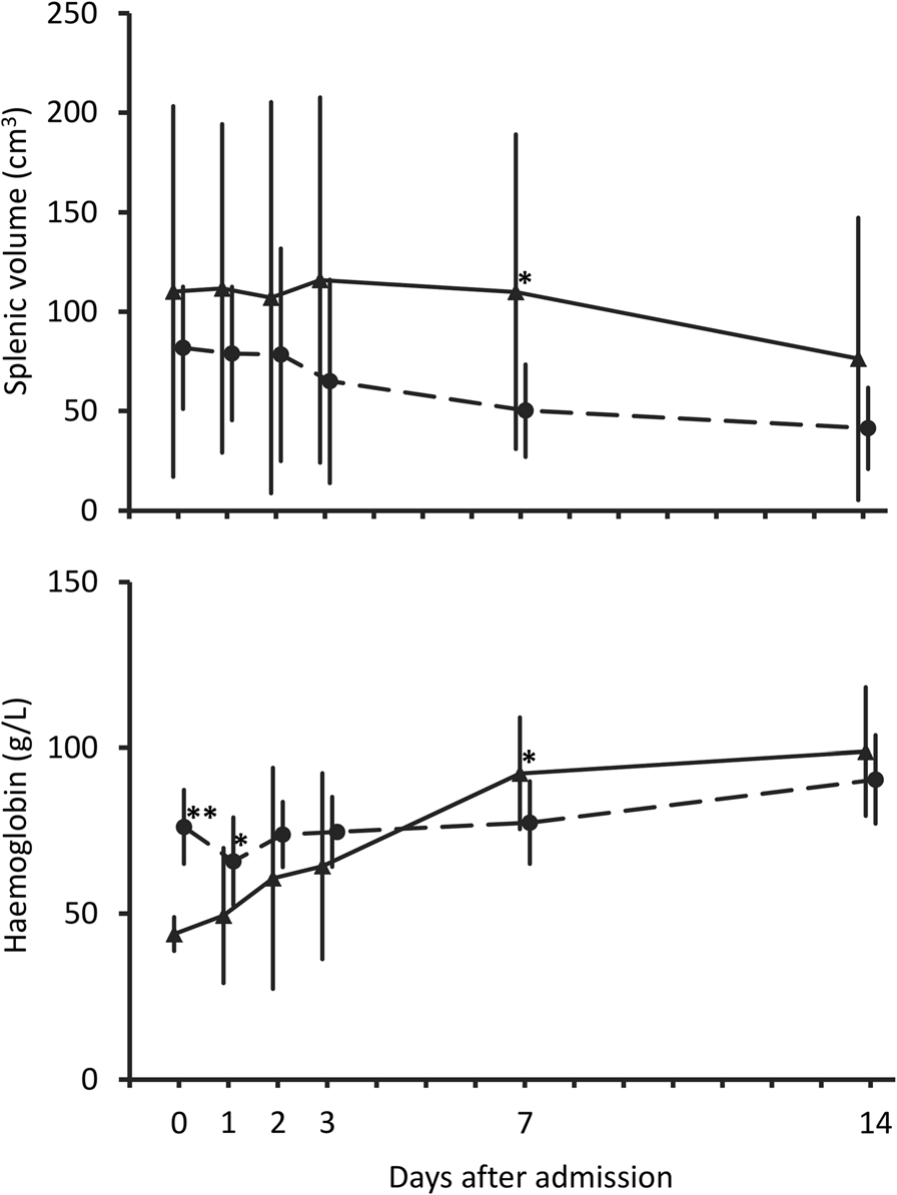
Serial changes in splenic volume (upper panel) and haemoglobin (lower panel) in children with MMA (●---●) compared to those with SMA who were transfused (▲––▲) Data points are means and SDs (vertical bars). *P <0.05 and **P <0.001 for between group comparison using generalized linear mixed-effects models

These were assessable in ten children with MMA and 12 with SMA with complete (including imputed) data to day 14. In children with MMA, mean splenic volume did not change initially but then fell after day 2 to approximately half the admission value at day 14 (*P* ≤0.011 *vs* days 0, 1 and 2; see Fig. 5). There was no equivalent change in the two weeks after treatment in the SMA group despite transfusion at around day 2 (*P* ≥0.30 for pair-wise comparisons between time-points to day 14). The mean splenic volume at day 7 was significantly higher than that in the MMA group at that time (*P* = 0.043). By day 14, there was no significant difference in splenic volume between the two groups of children, a situation that was also observed at days 28 and 42 (*P* >0.22).

There was no significant change in haemoglobin concentration in the two weeks after treatment in the MMA group (*P* ≥0.10 for pair-wise comparisons between time-points to day 14; see Fig. 5). There was, however, a significant rise in the relatively low haemoglobin concentrations in the SMA group after day 3 (*P* ≤0.05 for day 7 haemoglobin *vs* days 0, 1, 2 and 3; see Fig. 5), so that levels in the SMA group were higher than those in the MMA group at day 7 (*P* = 0.022). By day 14, there was no significant difference in haemoglobin between the two groups of children, a situation that was also observed at days 28 and 42 (*P* >0.38).

## Discussion

The present study demonstrates that splenic volume determined by ultrasound in children presenting with falciparum malaria in a hyperendemic area of coastal PNG is variable and not associated with the degree of anaemia at presentation. Simple clinical measures of splenic enlargement (uniaxial splenic length below the costal margin and Hackett’s grading) were only moderately associated with splenic volume in this patient group, consistent with the relatively low sensitivity and specificity of palpation compared with ultrasonography in ascertaining the presence and degree of splenomegaly regardless of the underlying cause [33]. The temporal changes in splenic volume after anti-malarial treatment differed by anaemia status. In SMA patients, blood transfusion given during the first four days of treatment was associated with a stable mean splenic volume over the first two weeks after treatment in the presence of a progressive rise in haemoglobin to day 7. By contrast, splenic volume decreased over the first two weeks after treatment in the MMA group without a significant change in haemoglobin. These observations suggest that transfusion attenuates resolution of malaria-associated splenomegaly but also that the spleen does not sequester a significant volume of transfused blood in children with malaria and severe anaemia.

Given malaria endemicity in the study area [34], the risk of other infections [35] and haemoglobinopathies such as Southeast Asian ovalocytosis which are relatively common in PNG [36], it is not surprising that most of the present children had a degree of splenomegaly when recruited and that this was not associated with features such as age and haemoglobin. Nevertheless, palpation with or without linear measurement of spleen size may be adequate for clinical studies and epidemiological surveys in which detection of splenic enlargement is important [2–4], as long as its limitations are acknowledged. Palpation for splenomegaly has a sensitivity of between 28 and 77 %, and a specificity of between 62 and 99 %, in comparison with diagnostic imaging modalities [33]. The greater specificity vs sensitivity is consistent with the fact that Hackett’s grade 0 was consistently associated with a normal splenic volume by ultrasound (<108 cm^3^) [32] in the present study. Ultrasonography represents a low-cost, non-invasive and accurate technique for repeated assessment of splenic volume [28–31, 33]. In the present study, novel ultrasonographic data were collected relating to temporal changes in this variable, which would not have been possible using simple clinical assessments.

The most interesting observations in the present study related to the relationship between serial ultrasonographic splenic volume estimates and haemoglobin concentrations after anti-malarial treatment. Reticuloendothelial clearance of parasitized and non-parasitized erythrocytes [7, 37] as well as acutely depressed erythropoiesis [10] can result in an initial temporary post-treatment reduction in haemoglobin [38]. This can be partially offset by splenic ‘pitting’, a process which is strongly related to artemisinin treatment and immune status [39] and which removes parasites from infected erythrocytes and returns these once infected cells to the circulation. In children with MMA in the present study, the initial fall in haemoglobin was not significant and splenic volume did not change during the first few days after anti-malarial treatment had been started. The later significant reduction in splenic volume was not associated with changes in haemoglobin in this group.

By contrast, the mean haemoglobin concentration in the SMA group increased between day 0 and day 7, reflecting transfusions given during the first four days [10]. A number of children with SMA had large spleens at baseline (>100 cm^3^ in one third). It has been shown that splenomegaly attenuates the efficacy of blood transfusion through splenic sequestration of erythrocytes [18], but this did not appear to be present in the present children since splenic volume did not increase significantly between days 0 and 3, and the rise in haemoglobin (mean change 48 g/L between days 0 and 7) was consistent with the volume of blood transfused (expected change 52 g/L for a 12-kg child receiving 300 mL packed cells [40]). However, there was also no reduction in splenic volume by day 7 post-treatment as was seen in the MMA group. This suggests that transfusion attenuates the resolution of malaria-associated splenomegaly, perhaps through mild or subclinical immune activation resulting from red cell incompatibility [41]. Alternatively, the children with SMA had a larger sequestered biomass of parasitized erythrocytes than those with MMA, which also contributed to persistent splenomegaly through host immune responses [42].

The present study had limitations. Determination of within- (intra-day and inter-day) and between-observer variation in ultrasonographic splenic measurements were considered but would have been difficult in a high-turnover paediatric ward and as part of home follow-up visits in the rural tropics. The number of trained operators both performing ultrasounds and assessing splenic size clinically was, however, restricted to limit measurement variability. There were relatively small numbers of children in each group but data collection over the six-week follow-up period was complete in most patients. A larger sample may have allowed an assessment of whether the inverse relationship between splenic volume and mortality found in an African paediatric study [16] is also a feature in PNG children but, consistent with the favourable outcome in all the present patients, the case fatality rate associated with severe malaria in Melanesian populations is relatively low [43].

## Conclusions

The present study demonstrates that ultrasonographic studies are feasible using a portable system in logistically challenging research environments. The recognized limitations of clinical assessment of splenic size were confirmed by the present data. Serial splenic volume measurements and simultaneous haemoglobin concentrations suggest that the spleen does not have a major role in determining changes in post-treatment haemoglobin, including in transfused patients with SMA.

## Abbreviations

ACT: Artemisinin combination therapy
D: Depth
L: Length
MMA: Moderate malarial anaemia
PNG: Papua New Guinea
r_s_: Spearman rank correlation co-efficient
SMA: Severe malarial anaemia
W: Width
WHO: World Health Organization
